# Favorable Outcomes After Arthroscopic Posterior Bankart Repair for Traumatic Posterior Shoulder Instability in Collision Athletes

**DOI:** 10.1016/j.asmr.2025.101264

**Published:** 2025-09-18

**Authors:** Daisuke Yamashita, Atushi Tasaki, Takayuki Oishi, Taiki Nozaki, Shota Mashimo, Nobuto Kitamura

**Affiliations:** a Department of Orthopedic Surgery, St. Luke’s International Hospital, Tokyo, Japan; b Department of Orthopaedic Surgery, Nippon Medical School, Japan; c Department of Radiology, Keio University School of Medicine, Japan; d Department of Rehabilitation, St Luke's International Hospital, Tokyo, Japan

## Abstract

**Purpose:**

To investigate the postoperative results of arthroscopic posterior Bankart repair for traumatic posterior shoulder instability in collision sports athletes and their clinical characteristics, including injury mechanism, symptoms, physical examination findings, and imaging features.

**Methods:**

Between January 2011 and April 2022, a retrospective review was conducted of collision-sport athletes who underwent arthroscopic posterior Bankart repair for traumatic posterior shoulder instability at a single institution. The inclusion criteria were posterior instability caused by trauma, absence of generalized joint laxity, and arthroscopic posterior Bankart repair. All the patients had a minimum follow-up of 24 months. Patient demographics, injury mechanisms, imaging findings (evaluated using radiographs, computed tomography, and magnetic resonance imaging, including posterior labral tears, posterior glenoid bone loss, glenoid retroversion, and reverse Hill-Sachs lesions), return-to-play rates, recurrence, and postoperative shoulder pain and Rowe score were evaluated. Pre- and postoperative Rowe scores were compared using the Wilcoxon signed-rank test. A *P* value of < .05 was considered statistically significant.

**Results:**

Of 517 shoulders operated on for instability, 21 (4.1%) had posterior instability. After excluding 8 cases, 17 shoulders from collision sports athletes were analyzed. The mean age of the athletes was 21.1 years. All patients had a positive posterior apprehension test, and 62% had a positive anterior apprehension test. Imaging revealed posterior glenoid bony defects in 85% of cases. The mean glenoid retroversion angle was 1.6° ± 3.6° as measured on axial computed tomography images. The mean follow-up period was 40.5 ± 22.9 months, and all patients returned to their preinjury level of sports activity at a mean of 6.5 ± 1.0 months postoperatively. At the time of injury, 6 patients experienced a posterior dislocation, one experienced a subluxation, and 6 reported only posterior shoulder pain. Before surgery, 7 patients had no history of complete dislocation, while 6 patients had recurrent dislocations (≥2 times). The mean interval from the first dislocation or symptom onset to surgery was 21.8 ± 20.8 months. The Rowe score improved significantly from 55 (range: 25-75) preoperatively to 95 (range: 50-100) postoperatively (*P* < .01). The minimum clinically important difference for the Rowe score was 13.4 points, and 92% of patients exceeded this threshold. However, one patient (8%) experienced redislocations, and 4 patients (31%) reported residual pain, with 3 of these cases involving glenoid cartilage lesions at the time of surgery. Although postoperative pain was more common in patients with cartilage lesions (60%) than in those without (13%), this difference did not reach statistical significance (*P* = .217).

**Conclusions:**

Arthroscopic posterior Bankart repair for traumatic posterior shoulder instability in collision sports athletes resulted in a low recurrence rate, high return-to-play rate, and clinically meaningful improvement. Although not statistically significant, residual postoperative pain tended to be more common in patients with glenoid cartilage lesions observed at the time of surgery.

**Level of Evidence:**

Level IV, retrospective therapeutic case series.

Posterior shoulder instability is estimated to occur in approximately 5% of all cases of shoulder instability.^1^^,^^2^ Reports from the United States indicate an incidence rate of 0.046 per 1000 person-year, compared with a greater incidence in studies within the U.S. military.^3^^,^^4^ It also has been reported that posterior shoulder instability and anterior shoulder instability differ in traumatic injury mechanism and that posterior instability is more common in women compared with anterior instability.^5^

Furthermore, the exact mechanism of posterior shoulder instability is often unclear,^6^ and in cases of high general laxity, minor triggers can lead to voluntary subluxation.^7^^,^^8^ Therefore, especially in the acute phase, the diagnosis and treatment strategy depend on the injury mechanism and the assessment of general laxity.^8^ Thus, the pathophysiology of posterior instability differs from that of anterior instability.

Reports to date on surgical outcomes for posterior shoulder instability are not limited to athletes but include patients at a wide range of activity levels, and joint laxity often contributes to posterior instability, even in the absence of a clear traumatic event.^9^, ^10^, ^11^ Similar to anterior instability, posterior shoulder instability caused by definite trauma is also common in collision sports athletes.^2^ Kim et al.^10^ reported on the surgical outcomes of arthroscopic posterior Bankart repair in patients with posterior shoulder instability, including 5 rugby players, one of whom retired after surgery. Nonetheless, clinical characteristics and treatment outcomes specific to collision athletes with traumatic posterior shoulder instability have not been thoroughly documented.

The purpose of this study was to investigate the postoperative results of arthroscopic posterior Bankart repair for traumatic posterior shoulder instability in collision sports athletes and their clinical characteristics, including injury mechanism, symptoms, physical examination findings, and imaging features. We hypothesized that arthroscopic posterior Bankart repair for traumatic posterior shoulder instability in collision sports athletes would result in favorable clinical outcomes with a low recurrence rate and high rate of return to sport.

## Methods

A retrospective study was conducted on collision sport athletes with traumatic posterior shoulder instability who underwent arthroscopic posterior Bankart repair at a single institution between January 2011 and April 2022, with a follow-up period of at least 2 years. Collision sports are defined as contact sports involving high-energy, intentional physical impact between players, such as rugby, American football, and wrestling. The study was conducted in accordance with the Declaration of Helsinki, and ethical review board approval and informed consent were obtained from all patients and relevant individuals before the study began (Research Ethics Committee of St. Luke’s International Hospital, Ref: 17-R036).

The inclusion criteria were as follows: (1) age 16 years or older, (2) no history of injury to the same shoulder, (3) injury caused by a clear traumatic event, (4) findings of posterior instability on physical examination (positive posterior apprehension test),^12^^,^^13^ and (5) cases with bony Bankart lesions or posterior glenoid rim erosion identified on preoperative computed tomography (CT), and clear posterior Bankart lesions confirmed on magnetic resonance imaging (MRI) or during arthroscopy, were included. Even in the absence of a clear traumatic dislocation as the initial event, patients with posterior shoulder pain and no clear history of dislocation or subluxation, but who showed posterior instability on physical examination and imaging findings (e.g., posterior apprehension test and posterior labral tear), were diagnosed with symptomatic posterior instability and underwent surgical repair. The exclusion criteria were as follows: (1) postoperative follow-up less than 2 years, (2) incomplete medical information, (3) high general laxity (Beighton score >4),^14^ (4) cases in which concurrent anterior Bankart repair was performed, (5) patients presenting with anterior instability on physical examination and exhibiting a definitive anterior Bankart lesion requiring repair during surgery, as these findings were indicative of multidirectional instability, and (6) lack of interest to participate in the study.

### Evaluation Items

**Fig 1:**
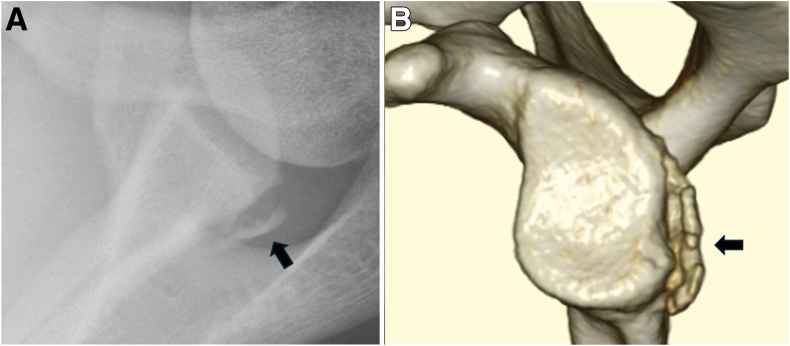
Image finding of the posterior bony Bankart lesion in the left shoulder. Arrows indicate the lesion. (A) Radiograph: Axillar view shows a posterior bony Bankart lesion. (B) Three-dimensional computed tomography view.

Age at surgery, sex, side of injury, sports, competition level, and injury mechanism were considered evaluation items. One experienced shoulder surgeon (A.T.) examined the preoperative physical findings, and posterior apprehension test, anterior apprehension test, and muscle strength (manual muscle) test were conducted.^15^ In the anterior and posterior apprehension tests, a positive finding was defined as either a reproducible sensation of apprehension (dislocation fear) or pain provoked by applied stress. We also investigated radiographs (anterior-posterior and axial views), 3-dimensional CT images, and noncontrast 3-Tesla MRIs. All the imaging evaluations were performed by a musculoskeletal radiologist (T.N.). On radiographs, the presence or absence of a bony Bankart lesion was assessed (Fig 1A). CT scans were used to evaluate glenoid bone defects, and the percentage of bone loss was measured as discussed by Sugaya (Fig 1B).^16^ The presence of Hill-Sachs lesions or reverse Hill-Sachs lesions also was assessed, and glenoid retroversion was measured. Glenoid version was measured using the glenoid vault method as discussed by Friedman.^17^ MRI T2∗-weighted axial images were used to evaluate labral pathology, including complete anterior-to-posterior detachment and incomplete tears such as Kim’s lesion.^18^^,^^19^

### Surgical Procedure

**Fig 2:**
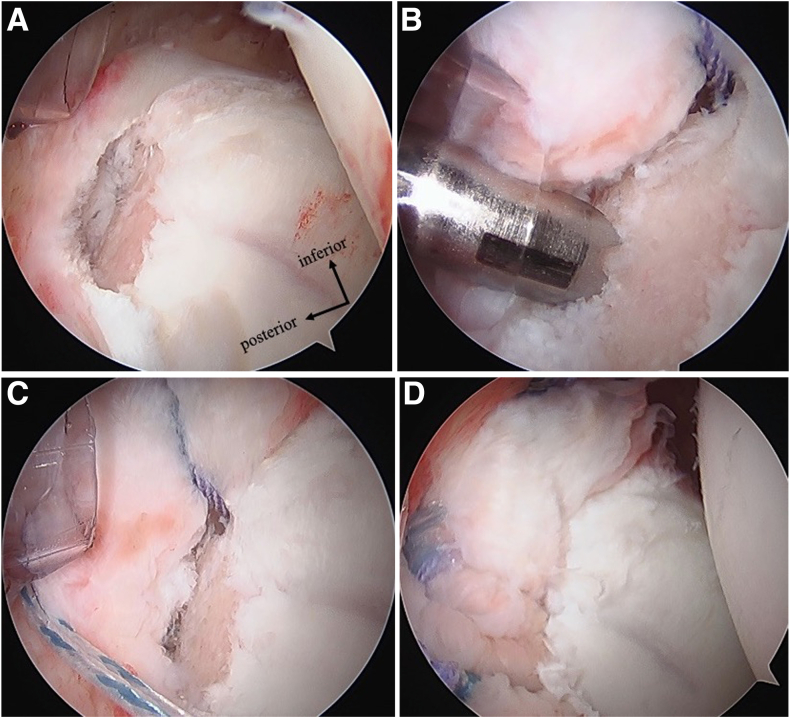
Arthroscopic surgical findings. The case involves traumatic posterior instability of the left shoulder with a bony Bankart lesion visualized arthroscopically through the anterosuperior portal. (A) A Bankart lesion with a bony defect is observed in the posteroinferior region. (B) An anchor is inserted into the glenoid neck as part of the double-row repair technique. (C) Sutures are passed through the bony lesion. (D) The sutures are tied with the anchors inserted at the glenoid edge.

All surgeries were performed by one experienced shoulder surgeon (A.T.) (Fig 2). The patient was placed in the lateral decubitus position with lateral traction of the affected arm at 40° abduction and 10° anterior flexion. Anterior, anterosuperior, and posterior portals were used; we observed from the posterior and anterosuperior portals. The damaged posterior capsulolabrum complex and bony Bankart lesion were repaired using a suture anchor. The suture method was simple suture or double-row repair.^20^^,^^21^ Rotator interval closure was performed in cases in which the surgeon identified significant attenuation or widening of the rotator interval. If a cartilage injury (glenoid labrum articular disruption [GLAD]) was present, a microfracture was performed depending on the defect size.

Surgical findings were evaluated the number of suture anchors and repair method (simple suture or double-row repair) and the clinical outcome (Rowe score) before and after surgery. The primary outcome variable was the change in the Rowe score from preoperative to postoperative evaluation.

Rehabilitation comprised a shoulder brace at 30° abduction and neutral rotation for the first 3 weeks postoperatively, followed by passive range of motion exercise. Patients began running at 8 weeks postoperatively, upper extremity strength training on the affected side at 10 weeks, skill exercises and weight training at 12 weeks, return to noncontact practice at 3 months postoperatively, and return to contact practice at 5 months postoperatively.

### Statistical Analysis

The minimum clinically important difference (MCID) for the Rowe score was calculated as 13.4 points, on the basis of one-half standard deviation of the change from preoperative to postoperative scores. Pre- and postoperative Rowe scores were compared using the Wilcoxon signed-rank sum test. The Fisher exact test was used to evaluate the association between intraoperative glenoid cartilage lesions and postoperative residual shoulder pain due to the small sample size. The significance level was set at *P* < .05. Statistical analysis was performed using the R software (Foundation for Statistical Computing).

## Results

Between January 2011 and April 2022, 517 shoulders were operated on for shoulder instability. Twenty-one shoulders (4.1%) of 20 collision sports athletes had traumatic posterior instability. One patient had bilateral injuries. After we excluded 1 patient who underwent concurrent rotator cuff repair, 1 with generalized joint laxity, 2 who were lost to follow-up due to relocation, and 4 patients who underwent concurrent anterior Bankart repair, 13 shoulders were included in the final analysis.

### Patient Demographics

All patients were male. The mean age at the time of surgery was 21.2 ± 4.0 years. The mean follow-up period was 40.5 ± 22.9 months, and the mean interval from injury to surgery was 21.8 ± 20.8 months (Table 1). The patient sample included 7 American football players, 5 rugby players and 1 martial arts athlete. All patients had a clear history of trauma during competition. Seven patients were injured by falling and hitting the ground with the shoulder, 3 patients were injured by forced horizontal extension, 2 patients were tackled and pushed backward, and 1 patient by forced horizontal flexion. At the time of injury, 6 patients experienced a posterior dislocation, 1 experienced a posterior subluxation, and 6 reported only posterior shoulder pain without overt instability. Three patients (23%) had experienced 2 to 5 dislocations, whereas another 3 patients (23%) had more than 5 dislocations. On physical examination, the posterior apprehension test was positive in all cases, with 7 cases of apprehension of dislocation and 6 cases of pain. The anterior apprehension test was positive in 8 patients (62%).

**Table 1:** Patient Demographics

Age, yr, mean ± SD (range)	21.2 ± 4.0 (18-32)
Mean follow-up, mo, mean ± SD (range)	40.5 ± 22.9 (24-83)
Duration from onset to surgery, mo, mean ± SD	21.8 ± 20.8
Time to return to sport, mo, mean ± SD	6.5 ± 1.0
Side, n (%)	
Right	5 (38%)
Left	8 (62%)
Sex, n (%)	
Male	13 (100%)
Female	0 (0%)
Competition level, n (%)	
Professional	2 (15%)
College	10 (77%)
High school	1 (8%)
Sport, n (%)	
American football	7 (54%)
Rugby	5 (38%)
Material arts	1 (8%)
Injury mechanism, n (%)	
Anterior shoulder impact	7 (54%)
Horizontal extension	3 (23%)
Posterior axial load during flexion	2 (15%)
Horizontal flexion	1 (8%)
Symptoms at onset, n (%)	
Dislocation	6 (46%)
Subluxation	1 (8%)
Pain	6 (46%)
Dislocation times, n (%)	
0 times	7 (54%)
1 times	0 (0%)
2-5 times	3 (23%)
>5 times	3 (23%)
Posterior apprehension test, n (%)	
Apprehension	7 (54%)
Pain	6 (46%)
Anterior apprehension test, n (%)	
Negative	5 (38%)
Positive	8 (62%)

SD, standard deviation.

Radiography showed bony Bankart lesions in 8 patients (Table 2), and CT showed bony defects in 11 patients (85%), with a median defect rate of 11.5% ± 7.5%. Bony Bankart lesions were seen in 11 patients (8585%), and Hill-Sachs lesions and reverse Hill-Sachs lesions were seen in 2 and 9 patients, respectively. The mean glenoid retroversion angle was 1.6° ± 3.6° as measured on axial CT images. Posterior complete labral detachment was identified in 9 patients (69%) on the basis of MRI.

**Table 2:** Imaging Findings

Radiography	
Posterior bony Bankart lesion, n (%)	8 (62%)
CT	
Glenoid bone loss	
Positive, n (%)	11 (85%)
Defect rate (%): mean ± SD	11.5 ± 7.5
Reverse Hill-Sachs lesion	
Positive, n (%)	9 (69%)
Hill-Sachs lesion	
Positive, n (%)	2 (15%)
Glenoid retroversion (°): mean ± SD	1.6 ± 3.6
MRI, n (%)	
Posterior labral complete detachment	9 (69%)
Posterior incomplete labral tear	4 (31%)
Partial detachment or irregular labral contour	3 (23%)
Reduced labral height	3 (23%)
Continuity between the labrum and glenoid is maintained	2 (15%)

CT, computed tomography; MRI, magnetic resonance imaging; SD, standard deviation.

Arthroscopic findings showed posterior Bankart lesions in all patients and posterior cartilage damage (GLAD) in 5 cases (Table 3). Of the 13 athletes, 5 underwent double-row posterior Bankart repair. The number of anchors used ranged from 2 to 4: 2 anchors were used in 1 patient (8%), 3 anchors in 6 patients (46%), and 4 anchors in 6 patients (46%). Rotator interval closure was performed in 2 cases, and microfracture for cartilage injury was performed in 2 cases.

**Table 3:** Surgical Procedure

Repair Technique	n	Additional Procedure	
		RI Closure	Microfracture to GLAD
Posterior simple repair	8 (62%)	1	1
Posterior dual-row repair	5 (38%)	1	1

GLAD, glenoid articular disruption; RI, rotator interval.

Two patients were aware of posterior shoulder pain at the time of return to competition; however, all patients returned to competition at a mean of 6.5 ± 1.0 months postoperatively and were able to play at the same level as before injury. They also showed improvement in the Rowe score from 55 (25-75) preoperatively to 95 (50-100) postoperatively. The 95% confidence interval for the median change was 30-70, and this change was statistically significant (*P* < .01). Twelve patients (92%) achieved an improvement in the Rowe score that exceeded the MCID threshold of 13.4 points.

One case (8%) of postoperative redislocation was observed. The degree of glenoid bone loss in that patient was 25.0%. Four patients (24%) complained of residual pain after returning to play. The degree of glenoid bone loss in these patients was 0%, 7.2%, 10.7%, and 11.1%, respectively. Three patients had a GLAD lesion. The incidence of postoperative pain tended to be higher in patients with cartilage lesions, the association was not statistically significant (odds ratio 10.5; *P* = .22) (Table 4).

**Table 4:** Patients With Poor Outcomes

No.	Age, yr	Residual Symptoms	Injury Manner	GLAD	Symptoms Just Before Return to Competition	Clinical Course
1	21	Posterior dislocation	Posterior axial load in flexion	Negative	None	Postoperative case of Bristow procedure at another hospital Treated conservatively
2	17	Pain	Horizontal extension	Positive	Pain	Continuing to play
3	20	Pain	Shoulder impact	Positive	Pain	Continuing to play
4	18	Pain	Shoulder impact	Negative	None	Continuing to play
5	20	Pain	Shoulder impact	Positive	None	Continuing to play

GLAD, glenoid articular disruption.

## Discussion

In this study, arthroscopic posterior Bankart repair in collision-sport athletes with traumatic posterior shoulder instability resulted in a high return-to-play rate, a low recurrence rate (8%), and clinically meaningful improvement in 92% of patients based on MCID analysis. Although not statistically significant, residual postoperative pain tended to be more common in patients with glenoid cartilage lesions observed at the time of surgery.

Posterior shoulder instability is a relatively rare condition, accounting for only 2% to 10% of all shoulder instability cases.^22^^,^^23^ Previous reports on posterior shoulder instability are limited, and most studies include heterogeneous patient populations comprising both non-traumatic cases with generalized joint laxity and traumatic posterior dislocations.^9^^,^^11^^,^^24^^,^^25^ As a result, the optimal surgical treatment strategy and prognostic indicators for posterior shoulder instability remain unclear.^26^ A systematic review by Gouveia et al.^22^ reported an average return-to-sport rate of 88% after surgical treatment of posterior shoulder instability, but only 68% of athletes returned to their pre-injury level, highlighting the considerable variability depending on injury mechanism and sport type. Rothrauff et al.^27^ found that more than 10 years after surgery, only 22% of patients continued to participate in their original sport, and 28% had completely retired from sport, underscoring the long-term challenges of achieving sustained return to sport. Notably, many of these studies included nonathletes and cases of multidirectional or nontraumatic instability, making it difficult to draw specific conclusions for collision athletes with traumatic posterior shoulder instability. The present study focuses exclusively on a homogeneous cohort of collision-sport athletes (e.g., rugby, American football, and martial arts) who sustained traumatic posterior shoulder instability and underwent arthroscopic posterior Bankart repair. Among 517 shoulder instability surgeries performed at our institution, only 13 patients (2.5%) met these criteria. Although the number of cases is limited, this study provides valuable data on a clearly defined and clinically relevant subgroup, which has rarely been addressed in previous literature.^28^^,^^29^

**Fig 3:**
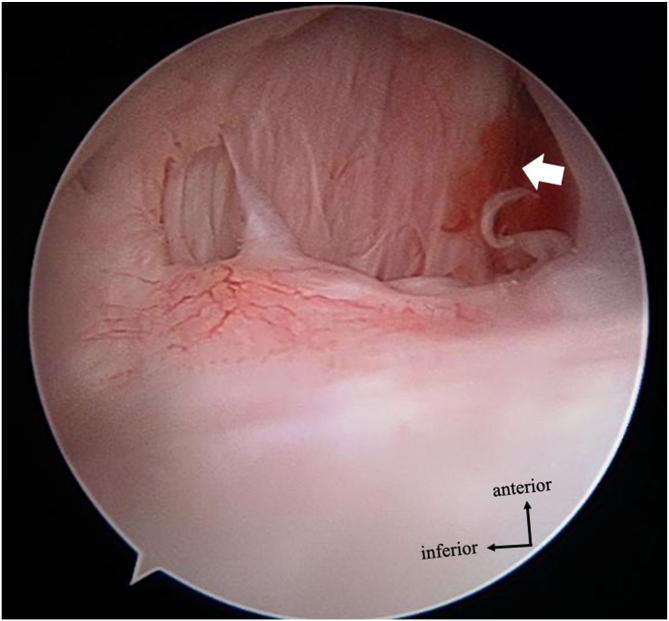
Arthroscopic findings during surgery for a case of redislocation (left shoulder, posterior view). The anterior articular capsule is absent, and the fibers of the subscapularis are partially exposed (indicated by arrow). This is presumed to be a consequence of the previous open Bristow procedure.

We could obtain a clear history of trauma in all cases; the onset of injury varied. In 6 cases, a sensation of dislocation/subluxation was not noted at the time of injury. Among them, 5 patients exhibited pain only during the posterior apprehension test without apprehension, whereas 1 patient showed a clear sense of posterior apprehension on physical examination. Imaging confirmed a posterior Bankart lesion in all 6 cases. Therefore, these cases were considered indicative of unstable painful shoulder.^30^ In terms of imaging studies, only 7 patients had clear findings of posterior labral complete detachment on noncontrast MRI, and seven patients had only findings of posterior incomplete labral tear. Labrum tear is often associated with minor changes on non-contrast MRI, such as loss of height and change in size.^18^^,^^31^ Therefore, the diagnosis of traumatic posterior instability must be comprehensively made based on the injury type, clinical findings, and imaging findings. However, CT examination revealed a high frequency of glenoid bone defects (11 cases, 85%) (Fig 3). Among these cases, only seven could be definitively diagnosed using plain radiography, highlighting the importance of 3-dimensional CT in evaluating bony changes and confirming the diagnosis of traumatic posterior instability. Reverse Hill-Sachs lesions were found in 9 cases (69%), which seems to be less common than Hill-Sachs lesions in anterior shoulder instability, and this finding, which is based on morphologic evaluation, may not be conclusive.^31^

The Circle Stability Concept is an important factor that contributes to shoulder joint stability.^32^ According to this concept, if instability occurs in either the anterior or posterior direction, similar symptoms and findings will also manifest on the opposite side. In this study, redislocation occurred in one cases. In one patient with a history of an open Bristow procedure, arthroscopic findings during posterior instability treatment revealed a defect in the anterior capsule. In such cases, even if an arthroscopic posterior Bankart repair is performed, the stability of the shoulder joint is likely to improve incompletely.^33^

**Fig 4:**
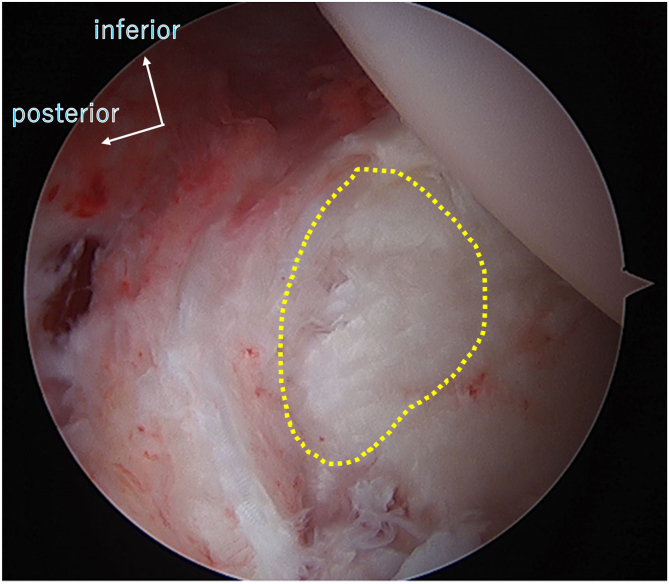
Glenoid cartilage lesion, left shoulder (anterosuperior portal view). A cartilage lesion is observed on the glenoid surface (outlined with a dotted line). Adjacent to this, damage to the capsulolabral complex is present.

In addition, residual pain was observed in 4 cases after return to competition. Cartilage lesions (GLAD) were found in 5 cases (Fig 4), and pain remained in three of them, suggesting that cartilage lesions may cause pain to recur postoperatively. Approximately 30% of postoperative patients with posterior instability have residual pain.^10^^,^^25^ This report did not mention the presence of cartilage damage; however, the results can be considered similar to our results. Although we attempted to repair cartilage defects using the microfracture technique, this may not be sufficient to resolve the problem. Furthermore, if the residual pain after surgery is a sequela of instability, augmentation techniques such as rotator interval closure, remplissage, glenoid bone grafting, or glenoid osteotomy^34^ may be effective in minimizing complaints.^35^ However, the development of osteoarthritis is another concern.

### Limitations

This study is not without limitations. First, it was a retrospective design, which inherently introduces the possibility of selection bias and limits causal inference. Second, it was a single-institutional study with a limited number of cases, and the choice of surgical procedure was at the surgeon's discretion including the indication for rotator interval closure, the number of anchors used, and the choice of repair method, all of which were based on subjective intraoperative judgment. Third, this study included only male athletes, and therefore, potential sex-based differences in the clinical outcomes could not be evaluated. Furthermore, variations in the fixation techniques (e.g., simple suture vs double-row repair) may have introduced heterogeneity in the outcomes. Pain symptoms were assessed on the basis of patient-reported discomfort during sports activity, and no validated pain scale, such as the VAS, was used. Range of motion and other patient-reported outcome measures were not systematically collected because of the retrospective design and inconsistent documentation in the medical records. Fourth, MRI arthrography was not performed in the current study. However, because MRI arthrography is not universally available in all facilities, we believe that the evaluation using noncontrast MRI in this study is clinically significant. Furthermore, the long-term outcomes of this surgical procedure have not been evaluated, and there is a possibility of a recurrence occurring later than 5 years postoperatively. Finally, there is no comparison with conservative treatment for traumatic posterior shoulder instability.

## Conclusions

Arthroscopic posterior Bankart repair for traumatic posterior shoulder instability in collision sports athletes resulted in a low recurrence rate, high return-to-play rate, and clinically meaningful improvement. Although not statistically significant, residual postoperative pain tended to be more common in patients with glenoid cartilage lesions observed at the time of surgery.

## Disclosures

All authors (D.Y., A.T., T.O., T.N., S.M., N.K.) declare that they have no known competing financial interests or personal relationships that could have appeared to influence the work reported in this paper.
